# Analysis of shared research data in Spanish scientific papers about COVID‐19: A first approach

**DOI:** 10.1002/asi.24716

**Published:** 2022-10-20

**Authors:** Roxana Cerda‐Cosme, Eva Méndez

**Affiliations:** ^1^ Universidad Carlos III de Madrid Madrid Spain; ^2^ Library and Information Science Department Universidad Carlos III de Madrid Madrid Spain

## Abstract

During the coronavirus pandemic, changes in the way science is done and shared occurred, which motivates meta‐research to help understand science communication in crises and improve its effectiveness. The objective is to study how many Spanish scientific papers on COVID‐19 published during 2020 share their research data. Qualitative and descriptive study applying nine attributes: (a) availability, (b) accessibility, (c) format, (d) licensing, (e) linkage, (f) funding, (g) editorial policy, (h) content, and (i) statistics. We analyzed 1,340 papers, 1,173 (87.5%) did not have research data. A total of 12.5% share their research data of which 2.1% share their data in repositories, 5% share their data through a simple request, 0.2% do not have permission to share their data, and 5.2% share their data as supplementary material. There is a small percentage that shares their research data; however, it demonstrates the researchers' poor knowledge on how to properly share their research data and their lack of knowledge on what is research data.

## 1 INTRODUCTION

Sharing and reusing research data is crucial for Open Science (OS) to be a reality. If research data cannot be found, accessed, integrated, and re‐used, the goals of OS will remain aspirations beyond the practical reach of many. Data management infrastructures, and openly published data, are necessary but not sufficient to achieve the promises of far more testing whether scientific results can be reproduced, and enabling wide use of scientific results to achieve both broader and faster innovation. In a recent publication, Ramachandran et al. (2021) highlighted the inherent collaborative and interdisciplinary nature of (Open) Science enabled by the technological developments accelerating scientific research and understanding, by empowering data and information‐sharing capabilities reaching not only the scientific community but the public at large.

OS is a movement but an attitude that “affects the way scientific work is carried out and is characterized by a broad collaborative effort at each stage of the research process” (Gagliardi et al., 2015). It is frequently presented as a cultural and systemic change that allows improving science through open and collaborative ways to produce and share research outputs, as early and open as possible during the research cycle. All the perceptions and the importance of OS in the world have been supported by UNESCO, where 193 countries approved the Open Science Recommendations at the end of November 2021,^1^ ratifying the need of OS after the COVID‐19 pandemic, including the importance of sharing research data.

The OS systemic change is understood as a transition from the traditional scientific communication paradigm based on the publication of papers to a new paradigm, which pursues the goal of opening up not only the publications themselves but also other research outputs like data, methodologies, software, etc. OS is the new paradigm for science and knowledge dissemination (Ardil, 2007; Kunst & Degkwitz, 2019; Smart et al., 2019) that fosters cooperative work and new ways of knowledge distribution by promoting effective data sharing (as early and broadly as possible) and a dynamic exchange of research outputs.

A scientific study suggested that research outputs (articles, books, and datasets) are growing by 8–9% a year (Bornmann & Mutz, 2015). During the COVID‐19 crisis papers, reports, preprints, and data have been published in record amounts that lead us to wonder how this information (data and publications) is being structured and validated so that it can be found, reused, accessible, and interoperable. “It is critical to have good quality data (including aspects of accessibility, timeliness and support for users, among others) for proper decision‐making” (Forero et al., 2021). We are witnessing an important change in the way of doing and sharing science: science in real‐time, which ignites OS but also needs meta‐research that helps to improve the effectiveness of scientific communication in crises such as the pandemic.

Most of the scientific papers on COVID‐19 are open access (Arrizabalaga et al., 2020; Belli et al., 2020) but “the real number of COVID‐19 datasets is hard to estimate […] and much data are not available on data repository because the research is still in progress or because of legal, industrial, or other reasons” (Azeroual & Schöpfel, 2021). In this study, we investigate how many of these datasets about coronavirus are really available, particularly those shared with COVID‐19 publications developed in Spanish institutions and, of course, if these datasets are open and FAIR (Findable, Accessible, Interoperable and Reusable) (Wilkinson et al., 2016) and the depository platforms or repositories follow TRUST principles (Transparency, Responsibility, User focus, Sustainability and Technology) (Corrado, 2019; Lin et al., 2020).

## 2 RESEARCH QUESTIONS AND OBJECTIVES

Different recent studies and reports have highlighted how the global public health crisis since January 2020 has changed the scholarly communication system (Waltman et al., 2021) “including our collective understanding of the vital role that data curation plays in scientific research, particularly viral outbreaks” (Shankar et al., 2021) and the dissemination of COVID‐19 research data underlying scientific publications (Gkiouras et al., 2020; Lucas‐Dominguez et al., 2021). In the survey conducted by RoRI (Research of Research Institute) of authors of COVID‐19 preprints, 47% of the respondents stated that they had made the data underlying their research publicly available; another 22% of the respondents stated that they had indicated in their preprint that data is available on request (Waltman et al., 2021). The last *State of Open Data 2021* report says that about a third of the respondents participating in the survey for this annual report indicated that they have reused openly accessible data more during the pandemic than before (Digital Science et al., 2021). With this perspective, the following research questions arises: Change to: How many research papers published by Spanish researchers about COVID‐19, have also shared their research data? And, if so, what are their attributes? Do research data deposited in repositories have the same attributes as research data associated as supplementary material? What are the requirements for accessing research data with and without permission to publish?

Despite the push for data sharing from manifestos,^2^ pledges,^3^ and calls,^4^ urged on by the COVID‐19 situation, data storage and publication are not yet implanted in the practice of current scientific communication, and many concepts are mixed or misleading. Our first review of data availability for research papers was pretty disappointing, since very few have linked research data in a proper repository. The expectations at the start of this study were low. Therefore, we wondered if the authors and editors recognize the difference between complementary material and data associated with the paper or research article.

The *general initial objective* is to study how many Spanish scientific papers on COVID‐19 published during 2020 share their research data. The following *specific objectives* take into account the limitations and likely methodology (see section 3): (a) Analyze the attributes of the research data deposited in repositories. (b) Verify the applicability of the same attributes for the analysis of the research data associated as complementary material to the papers. (c) Analyze the requirements for accessing the data associated with and without permission to publish the papers.

## 3 METHODOLOGY

### 3.1 Definition and scope of the object of study

The object of study is Spanish publications on COVID‐19 from January to December 2020. To select which publications should be included in our study, the papers published in journals indexed in Scopus database during 2020 were analyzed. This period was chosen because during 2020 two important events occurred that help us delimit the spectrum and time scope of the sample: on the one hand, the declaration of international emergency by the World Health Organization (WHO) and the development of the first and second waves in the European countries that unleashed the global growth rate of scientific publications, reaching 500 daily publications and 1,000 weekly publications (Torres‐Salinas, 2020).

### 3.2 Materials and resources

As basic material for the selection of the sample, Scopus database was chosen because of its coverage. Scopus combines “the characteristics of PubMed and Web of Science (WoS). These combined characteristics allow greater utility, both for research in the medical literature and for academic needs (citation analysis)” (Falagas et al., 2008).

The search sentence was:



TITLE‐ABS‐KEY ("2019‐nCoV" OR "COVID‐19" OR "SARS‐CoV‐2" OR "HCoV‐2019" OR "hcov" OR "NCOVID‐19" OR "severe acute respiratory syndrome coronavirus 2" OR "severe acute respiratory syndrome corona virus 2" OR "SARS‐CoV2" OR covid2019 OR "COVID‐19" OR covid19 OR 2019ncov OR "2019 ncov" OR "novel coronaviru*" OR "novel coronaviruses" OR "novel corona virus" OR "novel corona*" OR covid19 OR "covid 19" OR "sars cov 2" OR sars2 OR "new corona*" OR "new coronavirus" OR "coronavirus disease 2019" OR "coronavirus infection" OR "COVID‐19 illness" ) OR TITLE‐ABS‐KEY ( ( coronaviru* OR "corona viru*" OR "pneumonia viru*" OR cov OR ncov ) )






To limit the search, four filters were also applied: “Article” (as the type of document) “Spain” (as the author's country of affiliation), “2020” (as the year of publication). Only the articles whose status of publication was “final” and the subject area was “Medicine” were selected.



  LIMIT‐TO ( PUBSTAGE, "final" )



  LIMIT‐TO ( AFFILCOUNTRY, "Spain" )



  LIMIT‐TO ( PUBYEAR, 2020 )



  LIMIT‐TO ( DOCTYPE, "ar" )



  LIMIT‐TO ( SUBJAREA, "MEDI" )






The search was carried out on August 10, 2021, and 1,340 results were obtained. It was exported in comma‐separated values format (.CSV) according to Scopus' categories.

### 3.3 Analysis, methods, and limitations

When establishing a methodology that would allow us to consistently study the research data linked to the selected scientific publications, we encountered several limitations derived in general from the lack of consolidated standards and good practices in data sharing. The first limitation was detected when considering contrasting the data extracted from Scopus, with analogous data from Figshare and Zenodo. In the case of Figshare, the biggest problem was that this multi‐thematic repository does not have a mass‐download option. “Mass downloads cannot be made, and to consult the data, each item must be opened separately to download the files.” (communication by support@figshare.com, 2020). In the case of Zenodo, it allows the massive export of data in different formats, but when comparing both tables, a “key” or external data that could link both tables was not found, because the metadata assigned to the paper, and those assigned to the dataset, are not congruent.

We agree with the statement of Travieso Rodríguez and Ferreira Araújo (2019): “the availability of quantitative indicators from databases and information resources does not guarantee their global understanding, to which the particularities between scientific disciplines and the dispersion of the sources are added.” It follows that quantitative indicators are not enough to evaluate the research data shared in the papers of our study; it is necessary to establish qualitative indicators that help us know their nature.

In this sense, the second limitation detected was the absence of quality and impact indicators in research data evaluation scenarios such as ours. The measures or indicators for data are usually related to their openness, or availability of the data, rather than measuring their quality, impact, or reuse (Konkiel, 2020). This leads us to the first conclusion even before starting the study: There are different FAIR assessment tools,^5^ but the majority of the research datasets analyzed did not adhere to FAIR principles. A gap is evident within the construction of standardized criteria for the evaluation of research data. Furthermore, although the initial objective was also to analyze the “FAIRness” of the research data, this was impossible because of the lack of consistency of the data shared in the sample. Thus, the main method that we have adopted in this research is descriptive and detailed study based manually reviewing each of the papers and the dataset associated with them, which has been adopted in other studies that found the same limitation (Travieso Rodríguez & Ferreira Araújo, 2019; Vasilevsky et al., 2017).

The analysis was carried out in three stages that are described below:

1. In the first stage, the 1,340 papers were analyzed and categorized into those that did not have associated research data (1,173) and those that *somehow* had their research data associated (167).
2. In the second stage, the 167 papers were grouped according to how they shared data: (a) added as complementary material to the publication, (b) available upon request to the authors, and (c) uploaded to a data repository (Sixto‐Costoya et al., 2020).
3. In the third stage, different analysis criteria were applied to each group:
  - For the papers with research data deposited in repositories, the attributes proposed by Assante et al. (2016) and used in previous research (Travieso Rodríguez & Ferreira Araújo, 2019) were taken as a reference to construct the nine attributes (Table 1) that were identified and analyzed in the research data.
  - For papers with complementary material, five of the nine attributes were applied due to how they were shared.
  - Papers with associated data only available on request were grouped according to the reasons indicated in the declaration of data availability.

**TABLE 1:** Attributes for the evaluation of data deposited in repositories

N°	Attribute	Description	Source of analysis
(1)	Availability	Indicate whether the data associated with a dataset are publicly available or not	Public repository
(2)	Accessibility	The level of permanent accessibility to the dataset that guarantees access to the data	Persistent identifier (PID) assigned to the dataset
(3)	Format	Digital format according to the type of research data	Extension of the dataset file
(4)	License	Declaration of the license of the dataset and the policies governing the reuse of them, including access rights	Type of licenses associated with the dataset
(5)	Linkage	The datasets refer/connect to the paper and, in turn, the papers have the link to the shared research data	Ideally, a PID for the paper and another one for the paper
(6)	Financing	Indicate the agency that funded the project where the paper (and the data) are produced	Declaration of founder
(7)	Editorial policy	Indicate the journal policies on research data shared and associated with the paper established by the publisher	Published information about the journal rules regarding data sharing
(8)	Content	Type of content (subject, topic, etc.) of the research data associated with the paper	Declaration of the thematic coverage of the dataset
(9)	Statistics	Bibliometric and/or altimetric data associated with the datasets	Visualizations, downloads, citations, and/or alternative metrics

These nine attributes are a measurable alternative to FAIR (Findable, Accessible, Interoperable and Reusable). When FAIR principles are desirable for research data in the current research landscape of data sharing, they only “serve to guide data producers and publishers as they navigate around these obstacles, thereby helping to maximize the added‐value gained by contemporary, formal scholarly digital publishing” (Wilkinson et al., 2016). However, given the current poor state of data publishing and the technical complexity (metadata, machine‐readable, PIDs, vocabularies, and semantic artifacts, etc.) of making data FAIR we have applied these attributes (Table 1) that we have mapped to FAIR principles. In Table 2, we try to match FAIR data principles (Wilkinson et al., 2016), with the attributes selected for this study.

**TABLE 2:** Comparison of the FAIR principles with the attributes chosen for the dataset evaluation

Fair data guiding principles	Selected attributes for this study
Findable: The data and *metadata* can be found by the community after its publication, using search tools.	Accessibility (2) Availability (1)
F1. Assign the *(meta)data* a globally unique and persistent identifier F2. Describe the data with *rich metadata* F3. Register/index the *(meta)data* in a searchable resource F4. The *metadata* should clearly and explicitly include the identifier of the data described.	
Accessible: *(Meta)data* are accessible and can therefore be downloaded by other researchers using their identifiers	Accessibility (2)
A1. *(Meta)data* are retrievable by their identifiers using a standardized communications protocol A1.1. The protocols have to be open, free and universally implementable A1.2. The protocol must allow for an authentication and authorization procedure (where necessary) A2. The *metadata* must be accessible, even when the data are no longer available	
Interoperable: Both the data and the *metadata* should be described following the rules of the community, using open standards, in order to allow for their exchange and reuse	Content (8)
I1. *(Meta)data* must use a formal, accessible, shared and broadly applicable language for knowledge representation I2. *(Meta)data* use vocabularies that follow FAIR principles I3. *(Meta)data* include qualified references to other *(meta)data*	
Reusable: *(Meta)data* can be reused by other researchers, since their origin and conditions of reuse are clear	License (4) Format (3)
R1. *(Meta)data* have a plurality of accurate and relevant attributes R1.1. *(Meta)data* are released with a clear and accessible data usage license R1.2. *(Meta)data* are associated with information on their provenance R1.3. *(Meta)data* meet domain‐relevant community standards	

FAIR principles entail 80% metadata and vocabularies requirements (formal metadata describing data attributes, and “semantic artifacts”^6^) and 20% requirements for persistent identifiers (PIDs) (Table 2). However, there are neither formal metadata standards nor semantic artifacts universally adopted to check FAIR‐compliance. FAIR data principles focus on the technical components that would make a dataset findable, accessible, interoperable, or reusable, but they do not have any recommendation nor principle referring to the possible connection to a paper. Instead, we made a detailed manual check of the criteria/attributes included in Table 1. This qualitative analysis was possible due to the low number of publications in the sample that have shared their research data.

## 4 RESULTS AND DISCUSSION

**FIGURE 1:**
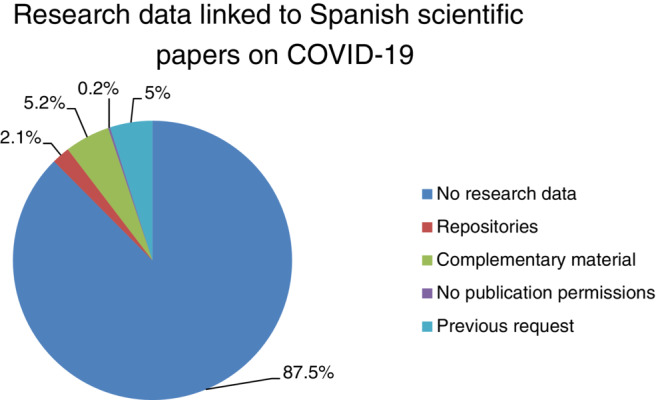
Research data linked to Spanish scientific papers on COVID‐19

As discussed in the methodology, to analyze the publication status of research data linked to Spanish scientific papers on COVID‐19, we analyzed the 12 months of 2020, selecting a final sample of 1,340 papers, where 1,173 (87.5%) did not have associated research data. The remaining 12.5% have the research data linked, in some way, to the publication: 28 (2.1%) papers published their research data in repositories, 3 (0.2%) do not have permission to publish the data, 66 (5%) will share the data upon request to the author, and 70 (5.2%) present the data (or other results) as “complementary material” (Figure 1).

We have limited our analysis to this 12.5% of the sample and divided it into the aforementioned three groups of scientific papers.

### 4.1 Scientific papers with research data deposited in repositories

#### 4.1.1 Availability (1)

**FIGURE 2:**
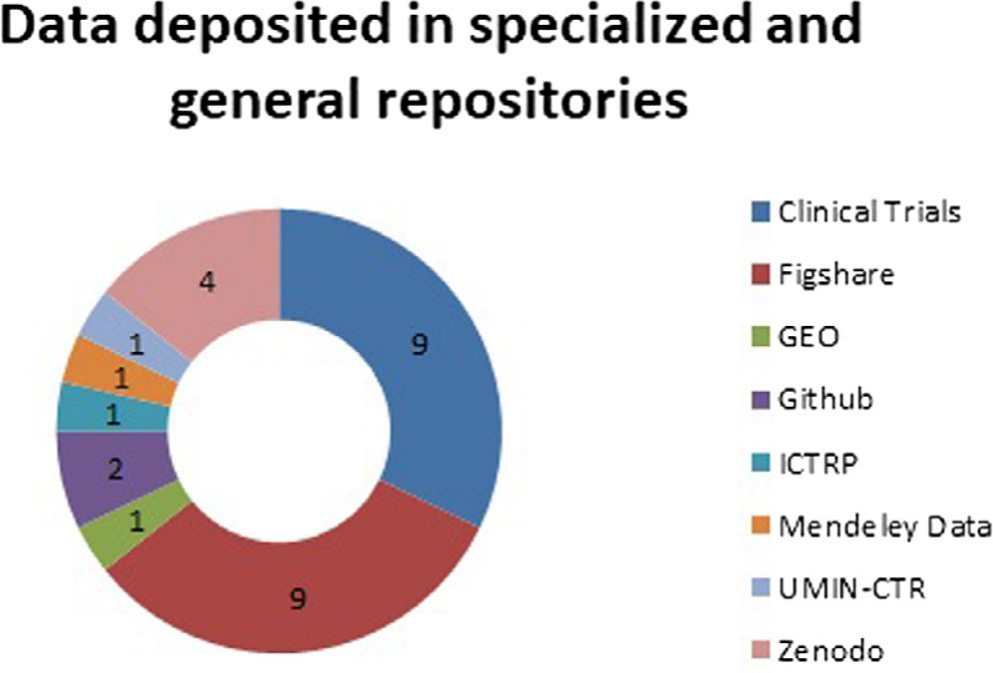
Data deposited in specialized and general repositories

The repositories that were used for the publication and availability of the data of the 28 papers identified in this group were either specialized, like ClinicalTrials or multi‐thematic, like Figshare or Zenodo. Figure 2 shows the number of papers that published their associated research data in each repository: ClinicalTrials (nine), Figshare (seven), Zenodo (four), GitHub (three), GEO (Gene Expression Omnibus) (one), ICTRP (International Clinical Trials Registry Platform) (one), Mendeley Data (one), UMIN‐CTR (University Hospital Medical Information Network—Clinical Trials Registry) (one).

The papers analyzed focused on medical aspects of COVID, so ClinicalTrials, GEO, ICTRP, and UMIN CTR are the repositories that were used to store clinical trials and genetic data. On the other hand, Figshare, Zenodo, GitHub, and Mendeley Data are multi‐thematic repositories that complement the clinical data deposited in specialized repositories, allowing its use in the development of other applications. These data were essential during the first and second waves of the pandemic in Spain, because there was limited knowledge of the virus and all the information that could be gathered about the cases and symptoms of patients helped the researchers.

#### 4.1.2 Accessibility (2) and linkage (5)

**FIGURE 3:**
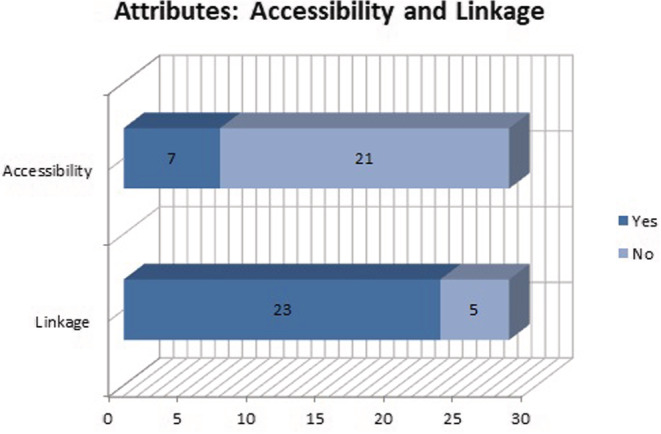
Data deposited in specialized and general repositories

The accessibility and linkage attributes were analyzed together. Because they are closely related characteristics to link a paper with its data, both resources must have a unique and PID. In the case of data: in 7 cases of the 28, they have assigned a DOI that identifies the dataset (Figure 3). The remaining 21 have an access link that is not a PID that can ensure their accessibility and durability. The link between the document/paper and its research data is fulfilled in 23 of the cases, while in five there is no link between data and paper.

Regarding accessibility and linkage, it is important to note that the existence of a PID is essential for the data to be considered FAIR (Table 2), specifically concerning location, availability, and accessibility, since having a Digital Object Identifier (DOI) enables permanent access and efficiently favors the localization of the datasets. Furthermore, it simplifies quoting the data, and thus promotes its reuse. We must not forget that one of the basic criteria when choosing a repository for research data is that it supports the assignment of DOIs for the data.

For the datasets published in a specific repository, they have their own DOI. According to Assante et al. (2016), citation is “a key mechanism in the publication of research data, as it allows data owners to obtain adequate recognition to publish their datasets and data consumers to explicitly refer to the datasets that are (re)used in their research.”

#### 4.1.3 Format (3)

The most used format corresponds to spreadsheets (.xls, .xlsx, .csv), followed by text formats (.txt, .pdf, .doc, .docx), structured (.xml, .R, .soft, .minimal), and visual (.tif) files. In the case of ClinicalTrials they can be downloaded in .pdf, .xml, .txt, .tsv, .csv and for GEO all files can be downloaded in a compressed .tar folder in .soft, .minimal, and .txt formats. In Mendeley Data, they were deposited in .xlsx. In Zenodo in .txt, .tsv, .csv, .tif. In Figshare in .docx, .xls, .pdf and in GitHub in .csv, .R, and in all cases one can download the compressed folder in .zip.

Likewise, data deposited in closed or proprietary formats such as .doc, .pdf, .xls are subject to the continuity of the associated software. It is good practice to use open formats produced by free software that allows for reuse.

#### 4.1.4 License (4)

Table 3 below collects the disparate casuistry of the licenses of the 28 datasets in repositories. It can be seen that data in Figshare and Mendeley Data use the Creative Commons Attribution 4.0 International (CC BY 4.0) licenses in all their consulted records, and for Zenodo in all but one of the records. This license allows the work to be distributed, mixed, adapted, even commercially, provided that the original creation is credited. ClinicalTrials, in its terms and conditions, states that the data deposited on this platform is in the public domain but requests that the source of information be recognized if the data are used or reproduced. GEO's disclaimer maintains that NCBI (National Center for Biotechnology Information), the institution responsible for the platform, does not impose restrictions on the use or distribution of the data; unless otherwise stated, documents and files on web servers are freely downloadable and reproducible. On GitHub, the first dataset does not indicate a license, the second uses the GNU Public License v3.0 which makes available the full source code of the licensed works and allows modifications, commercial use, modification, distribution, and use, while copyright notices, licenses, and status changes must be preserved. The third dataset applies the Apache 2.0 license which requires the preservation of copyright and license notices and allows commercial use, modification, distribution under different terms and without source code, free use of patents, and private use, but limits the use of trademarks. The International Clinical Trials Registry Platform (ICTRP) does not indicate the type of license it uses. According to Forero et al. (2021) CC0 license (universal public domain dedication^7^) should be used for data sharing.

**TABLE 3:** Licenses applied to data

Repository	Licenses				
	Public domain	CCBY40	Apache 2.0	GPL‐3.0	Not indicated
Clinical trials	✓				
Figshare		✓			
GEO	✓				
Github			✓	✓	✓
ICTRP					✓
Mendeley data		✓			
OSF	✓				
UMIN‐CTR		✓			
Zenodo		✓			✓

#### 4.1.5 Financing (6)

The sixth attribute studied is the funding agencies or organizations that supported the research. Fifteen of the 28 research works did not receive specific funding from any institution. In the sample of papers on COVID‐19, it was found that the Ministry of Science and Innovation financed three projects, two were carried out together with the Ministry of Education, Culture, and Sports, the Government of Catalonia, and the Ministry of Economy. The Alfonso Martín Escudero Foundation financed two projects. The Government of Aragon financed two projects, one of them with the Ministry of Economic Affairs and Digital Transformation. The Ministry of Education, Culture, and Sports financed two projects, both in conjunction with the *Junta de Extremadura*, the *Parque Científico de Madrid* Foundation, and the Ministry of Science and Innovation. The following institutions subsidized a project: Spanish Scientific Council (CSIC), Catalan Health Department, Interhospital Foundation for Cardiovascular Research (FIC), *Parque Científico de Madrid* Foundation, the Government of Catalonia, Bellvitge Biomedical Research Institute (IDIBELL), *Junta de Extremadura*, Ministry of Economic Affairs and Digital Transformation, and the *Universidad Autónoma de Madrid*.

#### 4.1.6 Editorial policy (7)

The seventh attribute is publishers' policies on the publication of research data linked to scientific papers. The publishing house Frontiers Media S.A. has seven papers with research data deposited in a repository. BioMed Central Ltd and Elsevier Inc. follow with four papers each; and MDPI AG with two papers.

Regarding research data policies, none establish the deposit of data as a mandatory requirement. Frontiers Media requires authors to make available the dataset that served as the basis for reaching the paper'

s conclusions. The data must apply the FAIR principles and they have a specialized area in data management that gives guidelines for citation, availability declaration, and deposit in repositories according to data type. Likewise, BioMed Central recommends that all datasets on which the conclusions of the document are based are available to readers. It provides a list of recommended public repositories organized by topic. All authors should include an “Availability of data and materials” section in their manuscript that details where the data supporting their findings can be found. Authors should also cite the publicly available dataset in their reference lists. It has a free advisory service operated by the Research Data Team. Elsevier also encourages researchers to publish their data and suggests free hosting at Mendeley Data. The data can be sent during the manuscript submission process or deposited directly; in both cases they will be assigned a DOI and a Creative Commons license. Three of the four publishers have information about their policies under different headings. An opposite case is the publisher MDPI AG: it does not have a research data policy.

Although publishers encourage researchers to publish their data, this does not mean that general repositories become the mixed bag of science, where documents that do not meet the definition of research data are deposited in any format without any restriction in this regard. In this sense, validation or verification from the editors or repositories of the datasets to which they give access is necessary to ensure that they are indeed original research data (Travieso Rodríguez & Ferreira Araújo, 2019).

#### 4.1.7 Content (8)

**FIGURE 4:**
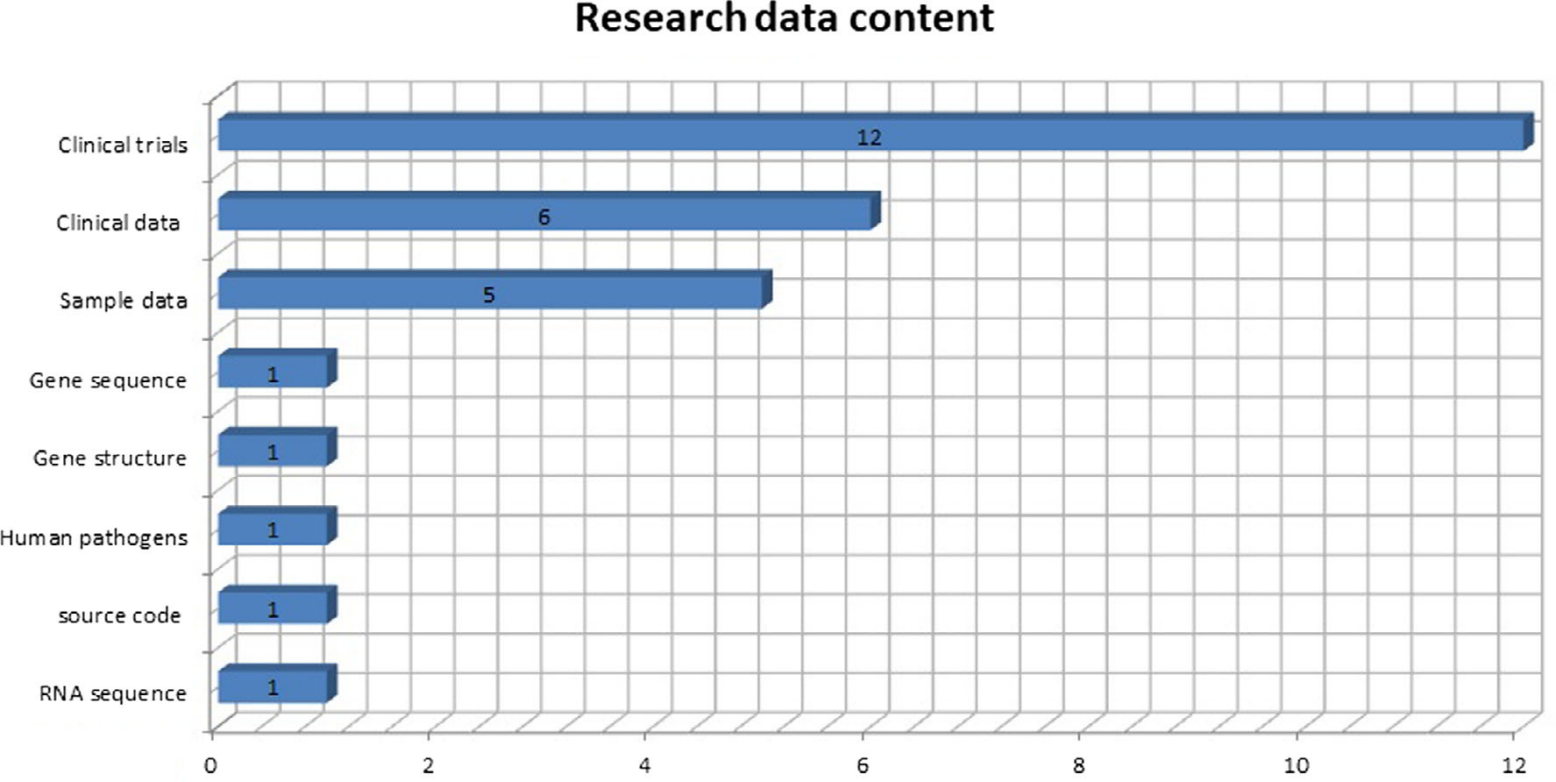
Research data content

In our sample, the content of the datasets responds to the nature of their paper. We identified 12 clinical trials, of which two have their final results published. One as complementary material to the paper and the other with the option of complete download in the deposited repository. The remaining 10 trials do not have their final results published. Six datasets contain clinical data and five with sample data. In both cases, patient data are anonymized to protect their privacy and avoid the identification of their personal data. Furthermore, four datasets contain information on the sequence of genes and RNA and one contains the source code in the R language of the developed application (Figure 4).

#### 4.1.8 Statistics (9)

They can be divided into two groups. First, there are the repositories such as Zenodo, Figshare, and Mendeley Data that include alternative metrics through Plum Analytics or Altmetrics as a complement to the statistics that the repositories contain, such as views and downloads. The second contains repositories such as GEO, ClinicalTrials, UMIN Clinical Trials Registry (UMIN‐CTR), International Clinical Trials Registry Platform (ICTRP), and Open Science Framework that do not specify information in this regard.

If we relate this attribute to the second accessibility attribute, it shows that assigning the DOI to citations increases the visibility of the data. The first step to promote the use of citations is to include them in the bibliographic references of the paper where the data are associated and add the DOI or the link that links them to the repositories where they are deposited. This practice helps to obtain citation statistics, which are complemented by the number of views, downloads, and alternative metrics.

In the context of the pandemic, our analysis of the statistics leads us to question for future research whether it was the data or the scientific papers that had the highest reuse rate in research related to an effective solution for the virus.

### 4.2 Papers with research data associated as a complementary material

The second group analyzed was the papers published with datasets as complementary material. 70 (5.2%) share their dataset as complementary data attached to the paper. For this group, we have applied five of the nine: format (3), license (4), financing (6), editorial policy (7), and content (8) were applied. Attributes availability (1), accessibility (2), linkage (5), and statistics (9) were excluded because they are not available in a repository. Therefore, their accessibility and linkage cannot be analyzed because they depend on the same DOI of the paper. This also affects the statistics because they might refer to the paper and not the dataset.

#### 4.2.1 Format (3)

Table 4 below shows that the most widely used formats in study are text (.txt .pdf .doc .docx .ppt) and audiovisual (.avi .flv .wmv .mp4 .jpg .png). The format and content attributes have a direct relationship, since the file format impacts the ability of current and future software to “import” the content of the dataset, while the content format impacts the interpretation and understanding of the dataset (Assante et al., 2016).

**TABLE 4:** Open data types and file formats

Type of data	Formats
Text	.txt .pdf .doc .docx .ppt
Spreadsheets	.xls .xlsx
Compressed	.zip .tar
Audiovisual	.avi .flv .wmv .mp4 .jpg .png

#### 4.2.2 License (4)

The predominant status of the papers of this group is “all rights reserved” (52). However, they are open access because of pressure from funders (e.g., Welcome Trust^8^), who asked researchers, journals, and funders to guarantee that research results, as well as relevant data on the virus outbreak, are shared quickly and openly to inform public health and ensure that the WHO has quick access to emerging findings, which could help save lives around the world for the duration of the coronavirus (COVID‐19) outbreak.

Creative Commons licenses apply to 18 papers. Twelve papers apply the CC BY 4.0 license that allows the distribution, mix, adaptation of the work, even commercially, so long as credit is given to the original creation. It is the most open license used for this group of datasets. Six apply the CC BY‐NC‐ND 4.0 type that only allows others to download the works and share them with other people, as long as their authorship is acknowledged, but they cannot be changed in any way or be commercially used. It is the most restrictive of the six Creative Commons licenses.

#### 4.2.3 Financing (6)

Of the 70 papers analyzed, 48 did not receive specific funding. The Carlos III Health Institute was the institution that provided the most funding, 11 research works benefited from the support. The Ministry of Science and Innovation is next with five research works.

#### 4.2.4 Editorial policy (7)

The largest number of publications with research data as complementary material is published by Elsevier with 26 papers, followed by MDPI with seven, Ediciones Doyma with six, Lancet and Springer with five, Oxford University Press with four, W.B. Saunders with three, American Medical Association and JMIR Publications Inc. with two. The publishers with one publication were: American Society of Hematology, BMJ Publishing Group, Churchill Livingstone, Cold Spring Harbor Laboratory Press, Frontiers Media SA, Massachusetts Medical Society, Mattioli 1885, Mosby Inc., Wiley‐Blackwell, and American Association for the Advancement of Science. Although publishers recommend the publication of the dataset in appropriate repositories, there is still evidence of a lack of advice to authors to identify and differentiate between a dataset and the complementary material of their research, and on good practices in the correct deposit of their data.

#### 4.2.5 Content (8)

**FIGURE 5:**
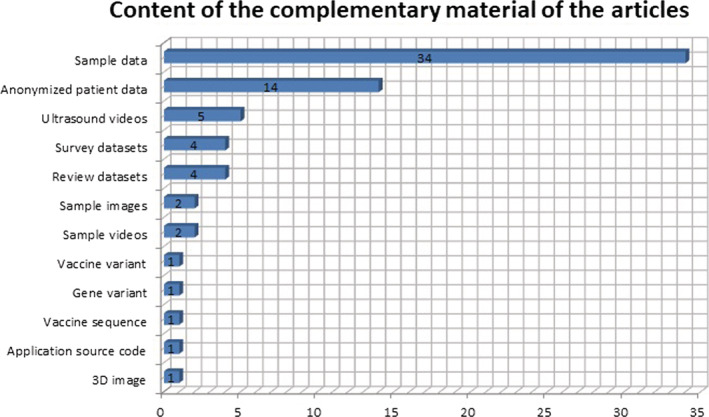
Content of the complementary material of the papers

The content analysis of the complementary material attached to the paper shows that it is research data (Figure 5). Thirty‐four sample datasets were identified, 14 are anonymized patient data, five ultrasound videos, four survey datasets, four review datasets, two sample images, two sample videos, one 3D image, one application source code, one vaccine sequence, one gene variant, and one vaccine variant. Figure 5 shows that only one part applies the concept of research data correctly and the rest tend to confuse it with complementary material. The authors declare and deposit as datasets materials that are not. Furthermore, papers were identified that indicate not having associated data, but when consulting the complementary material the dataset was available for download.

It is important to mention that there is a difference between the Supporting Information and the Data Availability Statement (DAS). The former brings together those materials that help to understand the paper more effectively, while the latter indicates whether there is research data to support the research. Some publications such as BMC y PLOS implemented a mandatory policy to include within their papers a DAS that obliges authors to indicate whether, where and how their data are available. A study conducted by Colavizza et al. (2020) states that the DAS is rapidly adopted after the introduction of a mandate in the journals from both publishers. For reasons largely related to what is proposed as a standard text for a DAS, BMC publications mostly use category 1 (data available on request), while PLOS publications mostly use category 2 (data contained within the paper and Supporting Information).

### 4.3 Papers with associated data with and without permission to publish

**FIGURE 6:**
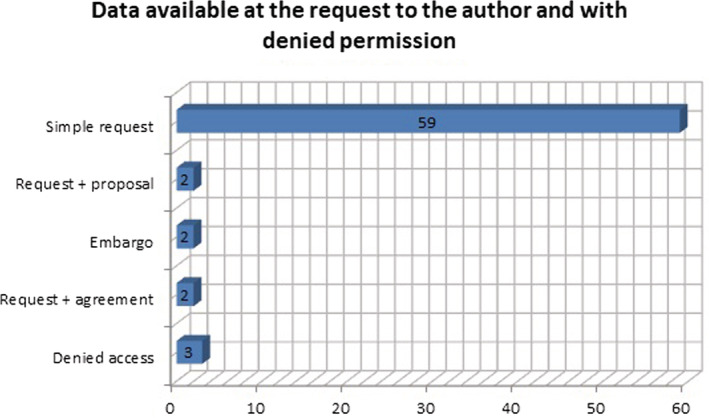
Data available at the request of the author and with denied permission

The third group analyzed was papers with data associated with and without permission for publication upon request. (Figure 6). Sixty‐six (5%) datasets were found that are available upon making a request to the authors, of which 59 are through a simple request, three request a detailed proposal with the study objectives, two a request and the signing of a data exchange agreement, and two have embargo dates; for the first paper the data will be available 18 months after the end of the trial and for the second paper the anonymized individual data will be available upon reasonable request during the first year after the publication of the main manuscript arising from the study. Three (0.2%) papers have denied access to their dataset because the first has confidential data, in the second the author does not have the permissions to share it, and the third shared the data in Google Drive and has access blocked.

The reasons that explain the results of our meta‐research involve the agents of scientific communication. The first relates to publishers and their research data management policies that suggest, encourage, and incentivize publication voluntarily, but is not a mandatory requirement. This does not mean that the data should be “open,” the applicable principle here is “as open as possible, as closed as necessary.” The data may have restricted permissions but its metadata should be Open Access, to help reuse in future research. The second is the reduced number of publishers and repositories that provide complete and detailed information on research data management and the implementation of a Data Management Plan (DMP). Researchers go first to publishers to be advised, but in some cases, the publisher does not have complete and up‐to‐date information or does not have trained staff. The third is incentives. Researchers do not receive an additional incentive for publishing their data. According to the type of research, the storage and preservation costs vary. These costs should be included in the initial project budget, but if a DMP was not developed or applied correctly, additional time and money must be invested in preparing the data so that it can be published.

## 5 CONCLUSIONS

After the study of its attributes, the limitations for the analysis of the datasets and the detailed analysis of the sample of Spanish publications on COVID‐19 and its shared data, the results answered the initial research questions and the proposed objectives were achieved. Furthermore, we can summarize several particular conclusions based on the findings of this work.

- Only 12.5% of Spanish scientific papers on COVID‐19 published during 2020 shared their research data and 87.5% have no associated research data, this is far from current global standards reflected in recent studies where over 48% of researchers acknowledge sharing data along with their publications on COVID‐19 (Waltman et al., 2021). There is an inconsistency between researchers' claims and their shared research data that highlights that assumption about shared data are not accurate.
- The attributes analyzed showed that there is a low awareness of how to properly share research data. Of the nine attributes, four are most notable for their lack of application: accessibility (2), linkage (5), format (3), and license (4). Datasets are not assigned a PID to be located and subsequently linked to their paper through citation. The choice of a free format to access the content and the type of license that indicates the limits of use are two attributes that are not yet assertively applied.
- The nine attributes cannot be applied to research data associated as supplementary material because, not being available in a repository, their accessibility and linkage is not evident due to the fact that they depend on the same DOI of the paper and the statistics refer to the paper and not to the dataset. Five attributes were applied: format (3), license (4), financing (6), editorial policy (7), and content (8). The most relevant is the content (8) attribute because papers were identified that indicate that they have no associated data, but when consulting the complementary material, the dataset available for downloading was found, showing that the authors declare and deposit as a dataset, materials that are not.
- The highest percentage of papers with data associated with permission to publish requested a simple request, while only 0.2% did not allow access to their datasets. Of the 12.5% of the papers that shared their data, only 0.2% denied or blocked access, this percentage confirms that researchers do not have knowledge about data anonymization, licensing, and data repositories. The first case does not share their data because it is confidential, in this case data anonymization could be applied in order to be able to share it. The second case does not have the permissions to share it and the third case shares their research data on an inappropriate platform such as Google Drive.
- With the results obtained we can affirm that there is a small percentage that shares their research data, however, the lack of knowledge about what is and what is not research data, which is demonstrated with attribute format (3) added to the incorrect way of sharing them, which is demonstrated with attributes accessibility (2), linkage (5), and license (4), proves that there is lack of knowledge and lack of training of the researchers.
- Our analysis proves that we are unlikely to achieve FAIR and TRUST until we can at least measure them and, preferably, make compliance more visible by integrating indicators into datasets and repositories in a way that is amenable to computational methods.

In general, the COVID‐19 pandemic caused an unprecedented increase in the number of publications, and also induces a change in traditional scientific communication, providing the necessary context for OS to be spread and developed. However, there is still a long way to go before the publication of data associated with a publication becomes standard practice and, even more so for its study to be systematized through the databases and repositories involved.

The lack of indicators for the evaluation of research data is evident. Lately, emphasis has been placed on creating regulations and digital verification and evaluation tools based on interoperability and reuse, leaving aside the indicators that describe the attributes and characteristics for qualitative evaluation of datasets.

## Supporting information


**Appendix S1.** Supporting Information.

## Data Availability

The datasets used for this study have been made available in Zenodo: Cosme‐Cerda, R., Méndez, E. *Analysis of underlying research data sharing in Spanish articles about Covid‐19 a first approach (January–December 2020)* Zenodo, November 18, 2021. https://doi.org/10.5281/zenodo.5711105

## Endnotes

^1^
UNESCO Open Science Recommendation (November 2021): https://unesdoc.unesco.org/ark:/48223/pf0000379949.locale=en

^2^
Manifesto of the European Commission “*Maximising the accessibility of research results in the fight against COVID‐19*” https://ec.europa.eu/info/research‐and‐innovation/research‐area/health‐research‐and‐innovation/coronavirus‐research‐and‐innovation/covid‐research‐manifesto_en

^3^
Open COVID Pledge, focusing in the Intellectual Property rights https://opencovidpledge.org/

^4^
The call on researchers by Welcome Trust *“Sharing research data and findings relevant to the novel coronavirus (COVID‐19) outbreak”*: https://wellcome.org/press-release/sharing-research-data-and-findings-relevant-novel-coronavirus-ncov-outbreak

^5^
Several tools (manual questionnaires, checklists and automated tests) that might be used to analyze the *FAIRness* of research data can be found in FAIRASSIST: https://fairassist.org however none of them were successfully applicable to the data‐sharing status on the studied publications.

^6^
Semantic artifacts are described by the FAIRsFAIR (https://www.fairsfair.eu) project as vocabularies for data descriptions: “the tools which allow humans and machines to locate, access and understand (meta)data” (Coen, 2019).

^7^

https://creativecommons.org/share-your-work/public-domain/cc0

^8^

https://wellcome.org/press‐release/sharing‐research‐data‐and‐findings‐relevant‐novel‐coronavirus‐ncov‐outbreak
